# Use of low-level laser therapy on children aged 1 to 5 years with energy-protein malnutrition: A clinical trial

**DOI:** 10.1097/MD.0000000000010538

**Published:** 2018-04-27

**Authors:** Karlla Almeida Vieira, Clarissa Moraes Bastos, Marilya Gabriella Correia Vitor, Alessandro Melo Deana, Kristianne Porta Santos Fernandes, Maria Fernanda Setúbal Destro Rodrigues, Vanessa Christina Santos Pavesi, Sandra Kalil Bussadori

**Affiliations:** a Department of Biophotonics, Nove de Julho University (UNINOVE), São Paulo-SP; b Faculty of Dentistry, CESMAC University Center, Maceió-AL, Brazil.

**Keywords:** child, lasers, malnutrition, saliva, salivary glands, semiconductor

## Abstract

**Background:**

Episodes of malnutrition in early childhood can produces alterations in the salivary glands. The investigation of mechanisms that can reduce the impact of malnutrition on the defenses of the organism is of the utmost important and interest to public health. The aim of this study is to evaluate the effect of low-level laser on the saliva of children aged 1 to 5 years with energy-protein malnutrition.

**Methods:**

Mandatory inclusion criteria are diagnosis of malnutrition. The sample will consist of 50 men and women malnourished children aged 12 to 71 months. Saliva will be collected and the volume of saliva will be measured and the salivary flow rate will be determined (mL/min). Concentrations of salivary IgA in all samples will be measured using a commercial Enzyme-Linked Immunosorbent Assay (ELISA) kit. Low-level laser (laser diode) will be administered in the region of the parotid glands bilaterally as well as in the regions of the submandibular and sublingual glands.

**Discussion:**

This study will be the first that investigate the effects of local laser therapy on the salivary glands of malnourished children.

**Trial registration:**

Clinical.trials.gov as NCT03355313, first received in 21 November 2017.

## 1 Background

Malnutrition is a clinical–social disease caused by multiple prenatal, intrauterine, and postnatal factors as well as social, political, and cultural determinants (distal causes).^[1]^ Despite the global and national reductions^[2]^ in the number of cases, malnutrition continues to be a public health problem, with greater prevalence in pockets of poverty found in the northern and northeastern regions of Brazil.^[3–6]^

Energy-protein malnutrition occurs when there is an imbalance between food intake and the physiological expenditure of energy and nutrients in all cells of the body, including those responsible for the formation of dental tissue, saliva, and the oral epithelium.^[7]^ Thus, malnutrition means that cells do not receive the nutrients necessary to perform their functions of energy production, tissue formation/repair, and the regulation of their own functioning.^[8,9]^

Malnutrition in the early years of life leads to growth deficits in childhood, as reflected by anthropometric indicators. This condition is also associated with a higher child mortality rate, greater probability of acquiring infectious diseases, impaired psychomotor development, and poorer academic performance as well as lower productivity in adulthood.^[10,11]^

Episodes of malnutrition in early childhood, with consequent calcium, phosphate, and vitamin A, C and D deficiencies, can increase one's susceptibility to dental caries through 3 probable mechanisms: defects in tooth formation (odontogenesis), delayed tooth eruption, and alterations in the salivary glands.^[12,13]^

It is likely that the significant increase in susceptibility to caries in malnourished individuals stems from alterations in the salivary secretion rate, since a reduction in salivary flow (salivary gland atrophy) increases the susceptibility to both dental caries and dental erosion.^[14–16]^ As saliva is the main defense factor of the oral cavity, a reduction/change in its physical properties (secretion rate and buffering capacity) can cause immunological disorders that affect an individual's defense capacity.^[17]^

Studies have demonstrated that salivary Immunoglobulin A (IgA) also plays an important role in the immunity of the oral mucosa.^[18–20]^ Indeed, patients with IgA deficiency can experience recurring upper airway (tonsillitis, ear infection, and sinusitis), lower airway (pneumonia), and gastrointestinal (diarrhea and parasitosis) infections.^[21]^

IgA was first identified in 1959 by Heremans^[22]^ and is found in serum and organic fluids, such as saliva. There are 2 types of this antibody. IgA1 is produced by B cells of the bone marrow and IgA2 is produced by B cells of the mucosa, accounting for approximately 80% of the total production. Once produced by plasmocytes (differentiated from activated B cells) in the lamina propria of the epithelium of the salivary glands, IgA binds to a polymeric Ig receptor in the base of the epithelial cells, where it is transported and released through proteolytic cleavage to join other substances that compose the salivary mucus.^[21,23]^

Stimulated and non-stimulated salivary flow rates can be reduced significantly in individuals who have suffered severe malnutrition in early childhood as well as those with continued nutritional stress. Thus, malnutrition can exert a continual effect on the reduction in salivary gland function in adolescence as result of malnutrition in early childhood, which suggests that exocrine systems can be compromised for long periods after exposure to malnutrition.^[24]^

The investigation of mechanisms that can reduce the impact of malnutrition on the defenses of the organism is of the utmost important and interest to public health. Among such mechanisms, low-level laser therapy has demonstrated effectiveness in the treatment of diverse conditions and disease through the promotion of the biomodulation of the cell metabolism and due to its analgesic and anti-inflammatory properties with no mutagenic or photothermal effects.^[25]^ The conversion of laser light into useful energy for cells through photochemical and photophysical reactions can simulate the production of mitochondrial adenosine triphosphate (ATP), cell proliferation, and protein synthesis.^[26,27]^

Laser stimulation of the major salivary glands to produce more saliva occurs through the increase in local circulation due to vasodilatation, the induction of the proliferation of glandular cells and cell respiration/ATP synthesis as well as the release of growth factors and cytokines to stimulate protein exocytosis.^[25]^ Regarding an increase in salivary IgA, low-level laser intensifies the activation of B lymphocytes, which differentiate into plasma cells, thereby contributing to the increase in immunoglobulin levels.^[28]^

It is important to study the impact of malnutrition in children because its long-term consequences could be reduced which implies promoting positive effects on oral and general healthy.

The primary aim of this trial is to evaluate the effect of low-level laser on the saliva of children aged 1 to 5 years with energy-protein malnutrition. Secondary outcomes are to establish the degree of malnutrition in children aged 1 to 5 years and to analyze the saliva of children aged 1 to 5 years with energy-protein malnutrition.

## 2 Methods/Design

### 2.1 Type of study

An experimental cross-sectional study is proposed, which will be conducted at the Center for Educational and Nutrition Recovery (CREN) in the city of Maceió, state of Alagoas, Brazil.

### 2.2 Trial registration

Clinical.trials.gov as NCT03355313, first received in 21 November 2017, https://clinicaltrials.gov/ct2/show/NCT03355313.

### 2.3 Sample

#### 2.3.1 Characteristics and size

This cross-sectional study will use a sample of children aged 12 to 71 months of the 7th Administrative Region of Maceió. This region is one of the poorest region in Maceió. The children's parents/guardians will be interviewed at the CREN in the city of Maceió, state of Alagoas, Brazil. The sample size was estimated considering a prevalence of malnutrition of about 10% in children under 6 years old, with statistical power of 80% and 95% significance, resulting in a sample of 50 children.

### 2.4 Allocation mechanism

The allocation sequence will be generated by CREN. The number/symbol of each child will be sent by the computer by the registration model in CREN which is based on the distribution by learning classes. Assessments regarding saliva and nutritional status will be blinding for trial participants and data analysts.

### 2.5 Inclusion criteria

Children aged 1 to 5 years enrolled at the CREN in the city of Maceió whose parent/guardians signed as statement of informed consent agreeing to the participation of the children.

### 2.6 Exclusion criteria

Children aged 1 to 5 years not enrolled at the CREN in the city of Maceió and children whose parents/guardians did not sign a statement of informed consent, teachers, caregivers, health professionals, and voluntary interns.

### 2.7 Interventions

#### 2.7.1 Evaluation of nutritional status

The children will be weighed on a duly calibrated electronic anthropometric scale (capacity: 150 kg; precision: 100 g) barefoot and wearing light clothing in the presence of the mother or caregiver. Height will be determined using a non-flexible metric tape measuring 2 m in length (precision: 0.1 cm). The measurement will be performed twice and the mean will be used for the calculation of the indices. Using these measures, height for age and weight for age will be determined using the nutritional reference standards recommended by the World Health Organization, with a cutoff point (*Z* score) between +1 and –1 standard deviations (SD) of the median indicative of the ideal range, *Z* between –1 and –2 SD indicatives of mild malnutrition, *Z* between –2 and –3 SD indicatives of moderate malnutrition and *Z* >–3 indicative of severe malnutrition.^[29]^

### 2.8 Saliva collection and analysis

Saliva will be collected at the dental sector of the CREN in the city of Maceió with the child seated in a dental chair under conventional light. Total resting saliva will be collected between 9 and 11 am (to avoid the influence of circadian rhythm) using the drainage method. The children will have not ingested food or beverages (except water) 1 hour before the procedure and will not have performed oral hygiene within 2 hours before the procedure. The child will be instructed to swallow prior to the collection and then instructed not to swallow, allowing the saliva to drain between the lips (which will be separated) into a test tube (aspirator) connected to a 15-mL Flacon tube positioned near the mouth.^[30]^ Collection time will be 5 minutes. The volume of saliva will be measured and the salivary flow rate will be determined (mL/min). Salivary flow values will be analyzed based on the values below^[31]^ (Table 1).

**Table 1:**
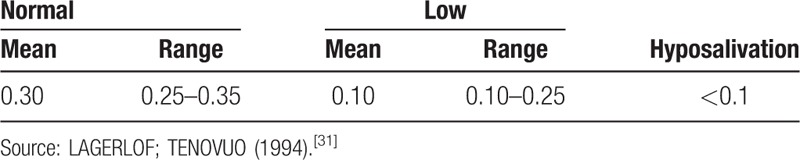
Salivary flow values for resting saliva (mL/min).

One aliquot (1 mL) of saliva will be transferred immediately to a small tube (capacity: 5 mL) for titration with hydrochloric acid 0.005 Ne. The tube will be shaken for 15 seconds and buffering capacity will be measured using a glass electrode (Orion) coupled to a pH meter (Procyon 720 A, Procyon, São Paulo, Brazil). Titration under partial carbon dioxide gas is considered the standard method for determining the buffering capacity of saliva. The reading of this test is performed based on the following parameters: pH 3.0 to 4.0 = very low to low buffering capacity; pH 4.5 to 5.0 = intermediate buffering capacity; and pH ≥5.5 = normal/good buffering capacity.^[32]^

**Figure 1:**
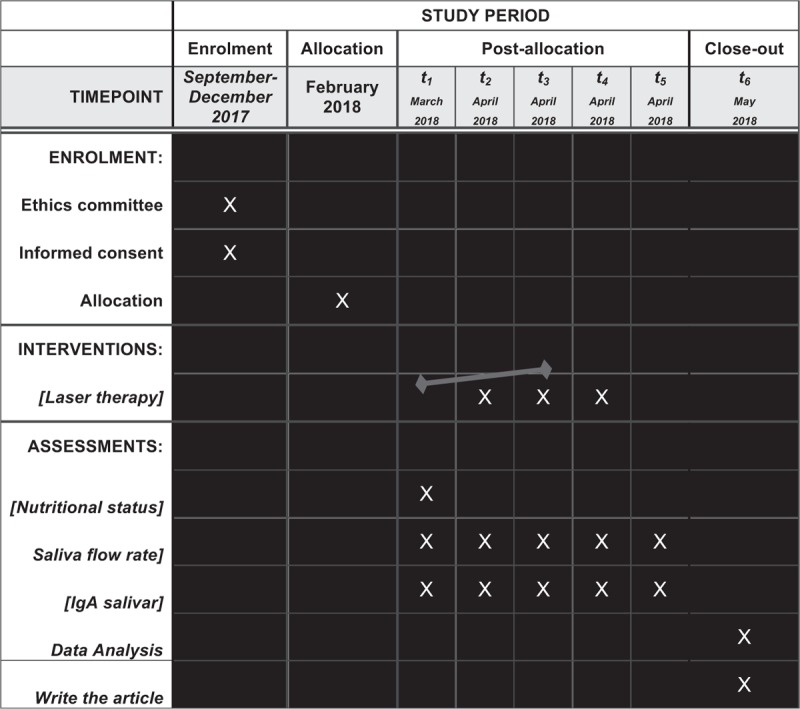
Schedule of enrolment, interventions, and assessments.

The remaining saliva will be stored in Eppendorf tubes and frozen at –20 °C until the analysis of salivary IgA. Concentrations of salivary IgA in all samples will be measured using a commercial Enzyme-Linked Immunosorbent Assay (ELISA) kit (Diametra IgA Saliva Kit, Italy) (Fig. 1). Salivary IgA (μg/mL) in each sample will be calculated using a standard curve obtained from the calibrators in the kit^[19]^ and will be analyzed^[33]^ (Table 2).

**Table 2:**
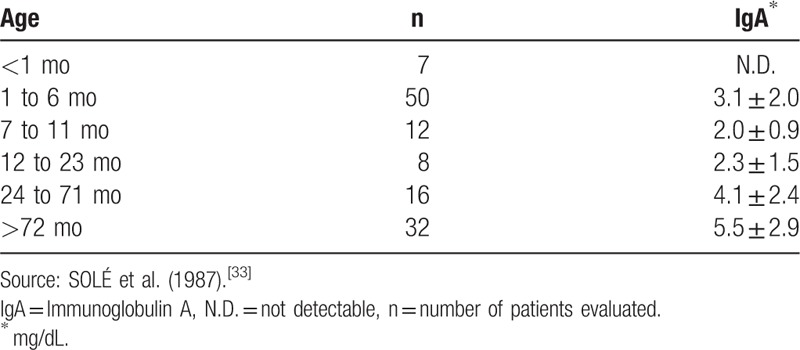
Normal IgA values in saliva of Brazilian children.

The data will be analyzed using Analysis of Variance (ANOVA) and Pearson's correlation test (*α* = 0.05). The Statistical Package for the Social Sciences (SPSS) (IBM Corp. Released 2012. IBM SPSS Statistics for Windows, Version 21.0. Armonk, NY: IBM Corp) version 15.0 will be used for all analyses.

### 2.9 Administration of low level laser

Low-level laser will be administered using the Photon Lase III device (DMC Equipamentos LTDA, São Paulo, Brazil). Laser will be administered for 10 seconds on 4 intraoral points and 4 extraoral points in the region of the parotid glands bilaterally as well as 1 intraoral point and 1 extraoral point in the regions of the submandibular and sublingual glands. The first session will occur after the collection of saliva. The second and third sessions will be performed 7 and 14 days after the first session, respectively. The final saliva collection will be performed after the third laser session.^[34,35]^

The laser will be adjusted according to the following parameters: Central wavelength (nm) = 808; spectral band width (FWHM) (nm) = 2; operational mode = continuous; mean radiant power (mW) = 100; polarization = randomized; aperture diameter (cm) = 0.2; irradiation at aperture (mW/cm^2^) = 2500; beam profile = multimodal; beam spot on target (cm^2^) = 0.04; irradiation at target (mW/cm^2^) = 2500; exposure time (s) = 40; Radiant exposure (J/cm^2^) = 100.0; radiant energy (J) = 4; number of points irradiated = 10; irradiated area (cm^2^) = 0.40; application method = Contact; number of treatment sessions = 3; frequency of treatment sessions per week = 1; Total radiant energy (J) = 40.

The salivary flow rate and salivary IgA will be compared before and after of each laser application (after 7 and 14 days) and correlation with the malnutrition will be established. The results will be analyzed using ANOVA and Tukey's contrast test. The level of significance will be 5%.

## 3 Conclusion

The study of salivary aspects in malnourished children and possible treatments that can be used to improve salivary quality and quantity in these children has significant social relevance, as saliva is one of the main mechanisms against infection and participates in essential functions of life, such as swallowing and the maintenance of oral health.

## Acknowledgments

Assistance provided by all the CREN employees was greatly appreciated. Also, the authors would like to show their gratitude to the children and parents.

## Author contributions

Conceive and design the study: Karlla Almeida Vieira, Alessandro Melo Deana, Sandra Kalil Bussadori. Will perform the experiment: Karlla Almeida Vieira, Clarissa Moraes Bastos, Marilya Gabriella Correia Vitor, Karlla Almeida Vieira, Maria Fernanda Setúbal Destro Rodrigues, Sandra Kali Bussadori. Will analyze the data: Karlla Almeida Vieira, Vanessa Christina Santos Pavesi, Maria Fernanda Setúbal Destro Rodrigues, Kristianne Porta Santos Fernandes. Will perform the statistical analysis: Alessandro Melo Deana, Sandra Kalil Bussadori. Write the paper: Karlla Almeida Vieira, Alessandro Melo Deana, Kristianne Porta Santos Fernandes, Sandra Kalil Bussadori.

**Conceptualization:** Karlla Almeida Vieira, Alessandro Melo Deana, Sandra Kalil Bussadori.

**Data curation:** Karlla Almeida Vieira, Kristianne Porta Santos Fernandes, Sandra Kalil Bussadori.

**Formal analysis:** Karlla Almeida Vieira, Alessandro Melo Deana, Maria Fernanda Setúbal Destro Rodrigues, Vanessa Christina Santos Pavesi, Sandra Kalil Bussadori.

**Investigation:** Karlla Almeida Vieira, Maria Fernanda Setúbal Destro Rodrigues.

**Methodology:** Karlla Almeida Vieira, Clarissa Moraes Bastos, Marilya Gabriella Correia Vitor, Alessandro Melo Deana, Maria Fernanda Setúbal Destro Rodrigues, Sandra Kalil Bussadori.

**Project administration:** Karlla Almeida Vieira, Sandra Kalil Bussadori.

**Supervision:** Karlla Almeida Vieira, Sandra Kalil Bussadori.

**Validation:** Karlla Almeida Vieira.

**Writing – original draft:** Karlla Almeida Vieira, Alessandro Melo Deana, Sandra Kalil Bussadori.

**Writing – review and editing:** Karlla Almeida Vieira, Alessandro Melo Deana, Kristianne Porta Santos Fernandes, Sandra Kalil Bussadori.
